# Vegetation Restoration Increases the Drought Risk on the Loess Plateau

**DOI:** 10.3390/plants13192735

**Published:** 2024-09-30

**Authors:** Hongfei Zhao, Jiaqi Dong, Yi Yang, Jie Zhao, Junhao He, Chao Yue

**Affiliations:** 1 College of Natural Resources and Environment, Northwest A&F University, Yangling 712100, China; zhaohongfei@nwafu.edu.cn (H.Z.); yangyi0712@nwafu.edu.cn (Y.Y.); 2 State Key Laboratory of Soil Erosion and Dryland Farming on the Loess Plateau, Northwest A&F University, Yangling 712100, China; jiaqikiki@126.com; 3 Shandong Provincial Key Laboratory of Water and Soil Conservation and Environmental Protection, College of Resources and Environment, Linyi University, Linyi 276000, China; sxuzhaojie@163.com

**Keywords:** revegetation, reclamation, hydrological effects, precipitation, soil water, Loess Plateau

## Abstract

The extensive implementation of the ‘Grain for Green’ project over the Loess Plateau has improved environmental quality. However, it has resulted in a greater consumption of soil water, and its overall hydrological effects remain highly controversial. Our study utilized a coupled land-atmosphere model to evaluate the effects of vegetation changes resulting from revegetation or reclamation on the hydrology of the Loess Plateau. Revegetation was found to stimulate an increase in precipitation, evapotranspiration, and atmospheric water content. However, the increase in precipitation was insufficient to compensate for soil water loss driven by intensified evapotranspiration, resulting in a decrease in both runoff and soil water content. In contrast to revegetation, reclamation would reduce precipitation, although the reduction was less than the decrease in evapotranspiration. This could lead to an increase in both runoff and soil water content. The results provide an important scientific basis for the hydrological effects of vegetation changes on the Loess Plateau, which is particularly important for guiding current and future revegetation activities toward sustainable ecosystem development and water resources management.

## 1. Introduction

China’s Loess Plateau (LP) is the largest and deepest loess deposit area in the world. With a precipitation (P) regime that includes extreme rainfall in summer, the loose loess deposit is prone to soil erosion, making the local environment fragile and vulnerable to human disturbance [1,2,3]. Indeed, intensified human exploitation over recent decades—for instance, the widespread construction of sloped farmland and over-grazing—has accelerated environmental degradation and threatened local sustainability [4]. In 1999, to try to halt soil erosion and improve the quality of the environment on the LP, the Chinese government launched the ‘Grain for Green’ program (GFGP), under which agricultural land on steep slopes was converted to forests and grasslands [5,6,7], a program considered to be the largest active revegetation program in China or elsewhere. Satellite observations over the early years of the 21st century show that the LP subsequently ranked as one of the most rapidly greening regions in the world [8], with its vegetation coverage more than doubling between 1999 and 2013 [6]. This substantial vegetation greening has improved the quality of the environment and curtailed soil erosion, significantly reducing both runoff and sediment from the LP [1,2,9].

However, such vegetation greening is accompanied by soil desiccation and plant mortality. Vegetation restoration has led to the consumption of more soil moisture than before the launch of the GFGP, rapidly depleting local water resources and leading to the formation of a dry soil layer [10,11,12]. The unsustainable water demand since the implementation of revegetation in 2000 has caused soil desiccation, which, in turn, has resulted in annual plant growth being water-limited and restricted by the current year’s rainfall and even vegetation degradation [13,14,15,16]. Accordingly, there are now serious concerns regarding whether re-vegetation is detrimental to water sustainability in the long term. Large-scale vegetation restoration can affect the exchanges of heat, water, and momentum by changing the land surface biogeophysical parameters, influencing boundary-layer processes, and even modifying large-scale atmospheric circulations, thus producing feedback effects on the regional climate [6,17,18,19,20,21,22,23,24]. Vegetation greening can facilitate surface actual evapotranspiration (ET) due to the larger leaf area, aerodynamically rougher surface, and higher canopy conductance for transpiration, causing a more humid atmosphere and thus increasing P [20,22,23,24,25,26]. But, if increased P cannot offset the water consumption caused by increased ET, the result will be a reduction of runoff and soil moisture, and, in arid regions, soil water resources will be jeopardized [27,28,29].

Afforestation can lead to an increase in ET [30,31,32], decreases in surface runoff and soil water storage [33,34], and perturb the hydraulic connectivity in dry regions [32,35]. However, previous studies have been unable to accurately evaluate the hydrological effects of vegetation restoration on the LP and have a low level of agreement on the outcomes. P has been shown to be increased by greening in most such studies [36,37,38,39] but is unchanged or decreased in others [18,40]. Even for the studies that report enhanced P due to greening, whether or not the P increase can compensate for ET-driven terrestrial water loss remains controversial [36,39,41]. Therefore, there is an urgent need for accurate and reliable research results regarding the hydrological effect of vegetation restoration on the LP. Such results will also help to answer the bigger question of vegetation greening’s effect in arid and semi-arid regions globally.

This study utilized a coupled land-atmosphere model to assess the impact of revegetation and reclamation on the hydrological processes of the LP. We seek to address the following questions: (1) What are the hydrological effects (P, ET, runoff, atmospheric water, and soil water content) of vegetation restoration over the LP? (2) Can the P increment compensate for ET-driven soil water loss? (3) What are the hydrological effects of reclamation in comparison to revegetation? Answering these questions will not only help address the uncertain ecohydrological effects of vegetation restoration over the LP but also provide insights for formulating further ecological restoration policy, both in China and other climatically similar regions of the world.

## 2. Materials and Methods

### 2.1. Study Area

The LP is located in the middle reaches of the Yellow River in north-central China (100°50′–114°32′ E, 33°42′–41°18′ N) and covers an area of around 640,000 km^2^. This region features a varied topography with altitudes ranging from 86 m to 4782 m and is primarily known for its loess deposits—among the largest and thickest in the world. The LP is located in a continental monsoon region and is characterized by a semi-humid and semi-arid climate, with annual P ranging from 110 mm in the northwest to 860 mm in the southeast and annual temperature ranging from 4 °C to 14 °C across the region [35]. Due to climate change and intensive human activities, serious soil erosion, coupled with the fragile ecological environment, makes the LP one of the most ecologically sensitive regions in the world [1]. In 1999, the Chinese government launched the GFGP, the largest active revegetation program ever attempted in China or any other country, with the aim of halting soil erosion and improving the ecological environment of the LP. Consequently, a large area of agricultural land on steep slopes has been converted to forests and grasslands [5,6].

### 2.2. WRF Model Configuration and Parameterization

To evaluate the effects of revegetation and reclamation on the hydrological processes of the LP, we use the Weather Research and Forecasting Model (WRF, version 3.9.1.1) [42], a fully coupled land-atmosphere regional climate model. The WRF model has been widely adopted to assess the effects of vegetation changes on surface hydrological processes at regional scales [18,22,40,41].

**Figure 1:**
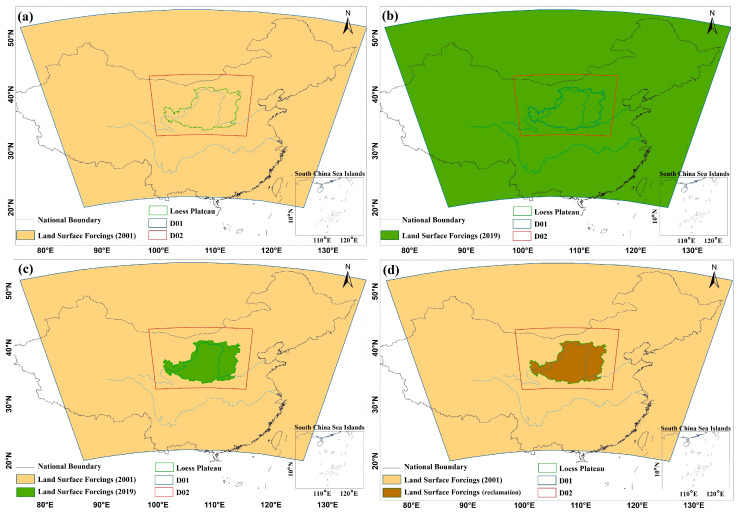
The experimental design of the WRF simulation. The outer domain (D01) and the inner domain (D02) are configured for all experiments. The experimental design of (**a**) EX_2001_, (**b**) EX_2019_, (**c**) EX_2019_S,_ and (**d**) EX_reclamation_.

WRF was configured with two nested, two-way interactive domains with spatial resolutions of 18 km and 6 km, respectively (Figure 1a). Specifically, the outer domain (D01), covering almost all of China, consists of 100 × 100 grid points with a spatial resolution of 18 km, which enables the climate impacts of topography and monsoon dynamics on the LP to be captured [43]. The inner domain (D02), which covers the LP region with 151 × 151 grid points and has a spatial resolution of 6 km, allows more refined simulations of the regional climate on the LP. The vertical layers were configured with 33 levels for both domains, with the atmospheric pressure of the topmost layer being set at 50 hPa.

The Noah land surface model (LSM), which describes land-atmosphere exchanges and transports of heat and water from the surface to the atmosphere [44], was coupled with the WRF model to provide the lower boundary conditions over land. In complicated terrains, such as the LP, a reliable parameterization of land-atmosphere interactions is very important to raise the skill level of the models [45]. Optimal parameterizations for the LP for various physical schemes were selected following previous studies [39,41,46,47] (Table S1). These consist of the Kain-Fritsch cumulus scheme [48], the Dudhia shortwave radiation scheme [49], the Rapid Radiative Transfer Model (RRTM) for longwave radiation [50], the CAM5.1 microphysics [51], MYNN2.5 planetary boundary [52], and the revised MM5 Monin-Obukhov surface layer scheme [53].

Initial and lateral boundary conditions with a 6-h temporal resolution were obtained from the Global Final Analysis data provided by the National Centers for Environmental Prediction (NCEP-FNL, http://rda.ucar.edu/). This is the most widely used source of data for the mesoscale model initial and boundary conditions. NCEP-FNL data are available on pairs of 1-degree hemispheric grids (north and south) at the Earth’s surface, with twenty-six vertical levels from 1000 millibars up to 10 millibars, the tropopause, boundary layer, two subsurface levels, and a few others. The parameters include surface pressure, sea level pressure, geopotential height, temperature, relative humidity, snow depth, precipitable water, wind, and others.

### 2.3. Experimental Design

The effects of vegetation restoration on the hydrology of the LP can be evaluated by comparing the changes of hydrological components (i.e., precipitation, runoff, evapotranspiration, soil water content) during the initialization of the GFGP and after the vegetation has attained a relatively stable state. Based on the comprehensive land surface forcing data and the actual situation in the LP, the year 2001 is defined as the start time of revegetation, while 2019 marks the point at which the vegetation has attained a relatively stable state. Any bias introduced by the 1-year gap between 1999 (the launch of the GFGP) and 2001 (MODIS land cover distribution data start time) is assumed to be small compared with the changes in land cover type and land surface biogeophysical parameters between 1999 and the present [40]. Accordingly, we conducted two numerical experiments: (1) the control experiment (EX_2001_) and (2) a sensitivity experiment (EX_2019_S_); the details are in Figure 1 and Table S2. The land forcings, including land cover, ground vegetation fraction (i.e., ‘VEGFRA’ in WRF terminology), leaf area index (LAI), and surface albedo, for the corresponding years of 2001 and 2019 were applied to the two simulation domains (D01 and D02), respectively, for EX_2001_ and EX_2019_S_ (data sources are detailed below). The changes in hydrology over the LP due to vegetation restoration efforts are then revealed by the difference between the results of EX_2001_ and EX_2019_S_.

Previous studies utilized the 2019 land use data over the entire spatial domain to set up the forcing in the sensitivity experiment and could, thereby, incorporate the nonlocal effects of vegetation changes in the surrounding area of the LP [18,39,40,46,47]. To confirm the existence of such nonlocal effects, we performed an additional sensitivity experiment, EX_2019_, in which the 2019 land use data was applied to the entire outer domain (D01), while all other forcings were identical to those applied in EX_2019_S_ (Figure 1 and Table S2). The difference between EX_2019_ and EX_2019_S_ then quantifies the nonlocal effects that vegetation changes in the outer region of the LP exert on the hydrology processes of the LP. The results show that the vegetation changes outside the LP resulted in a reduction in annual P in the LP region, which may have led to previous studies underestimating the impact of vegetation restoration on P. However, this reduction was a consequence of altered seasonal P patterns, with increased P in summer and decreased P in other seasons, compared with the patterns obtained in EX_2019_S_ (Figure S1).

Although vegetation restoration improves the quality of the environment and halts soil erosion in the LP, vegetation greening is accompanied by soil desiccation and plant mortality. Numerous studies have shown that forests consume more soil moisture than crops do, thus rapidly depleting local soil water resources and leading to the formation of a dry soil layer [1,54,55,56]. With the aggravation of the soil drying problem on the LP, an increasing number of scientists are questioning the effectiveness of GFGP. If the policy of deforestation for farming (Reclamation) is implemented instead of GFGP, what impact would the deforestation for farming policy exert on the hydrological processes in the LP? To investigate this question, we conducted another sensitivity experiment (EX_reclamation_) to implement the “Green for Grain” program (Figure 1 and Table S2). The difference between EX_2001_ and EX_reclamation_ provides the simulated hydrological effects of land reclamation compared to GFGP. The “deforestation for farming” model simulation, EX_reclamation_, requires the generation of a new land surface forcings dataset. To create this, we established a search window of 10 km by 10 km centered around each forest or grassland pixel for which the topographical falling gradient is less than 15 degrees. We assigned the average values of key land surface parameters (land cover distribution, ground vegetation fraction, LAI, and surface albedo) of the cultivated land pixels in each window to the central pixel (Figure S2). The value of the central pixel can thus be calculated using Equation (1).

(1) $${Value}_{central\;pixel}=\frac{{Value}_{{crop}_{1}}+{Value}_{{crop}_{2}}+\ldots \ldots +{Value}_{{crop}_{n}}}{n}$$

where *n* represents the number of cultivated land pixels in each window.

### 2.4. Datasets Used

The land surface forcings consisted of land cover distribution, ground vegetation fraction (i.e., the‘VEGFRA’in WRF terminology), Leaf Area Index (LAI), and surface albedo. For land cover distribution, the MODIS MCD12Q1 version 6 product was used. This provides global land cover distributions at a 500 m spatial resolution for 2001–2020. The ground vegetation fraction was approximated using the fraction of photosynthetically active radiation (FPAR) following the previous practice [57,58]. Both the FPAR and LAI datasets were from the MODIS MCD15A2H version 6 product covering 2001–2020 with a 500-m spatial resolution. Surface albedo was from the daily MODIS MCD43C3 version 6 product with a 0.05° spatial resolution. We used the average of black- and white-sky albedos for shortwave radiation at local noon. In order to be used in the WRF model, the 8-day LAI, FPAR, and daily albedo data were composited into a monthly time step.

The dataset of daily precipitation products used to evaluate the WRF model was retrieved from the China Meteorological Forcing Dataset (CMFD), which covers 1997–2018 at 10-km spatial and 3-h temporal resolutions. This dataset was produced specifically for China by combining ground-based meteorological observations from more than 1000 stations across the country with five remote sensing and reanalysis products [59].

### 2.5. Model Evaluation

To evaluate the WRF model, the simulated P over the LP was compared with the observations from CMFD. The results confirmed WRF’s capability to reproduce the seasonal variations of observed P well (Figure S3). The goodness of fit (R^2^) for the linear regressions between the simulated and the observed P was 0.77.

## 3. Results

**Figure 2:**
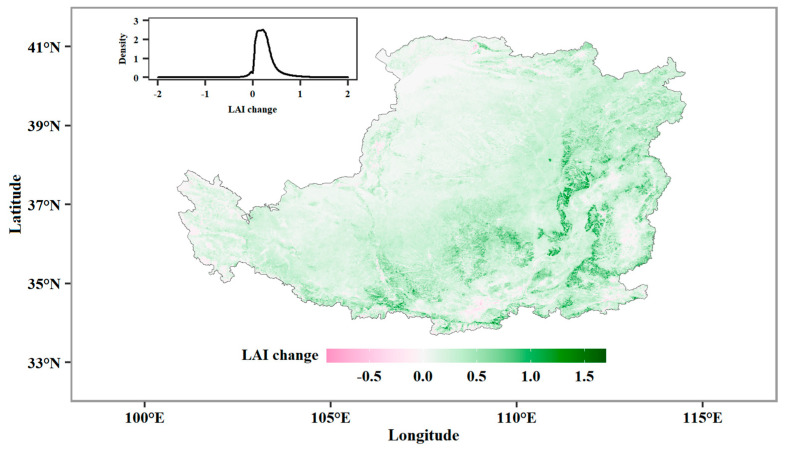
The change in vegetation LAI between 2001 and 2019. The inset plot in the panel shows the probability density distribution for ∆LAI.

The vegetation has been markedly restored since the launch of the GFGP. Compared with 2001, 96.6% of the LP had a greening trend in 2019, with the greening being particularly marked in the east and south of the LP and less pronounced in the northwestern and western parts (Figure 2).

### 3.1. The Hydrological Effects of Revegetation

**Figure 3:**
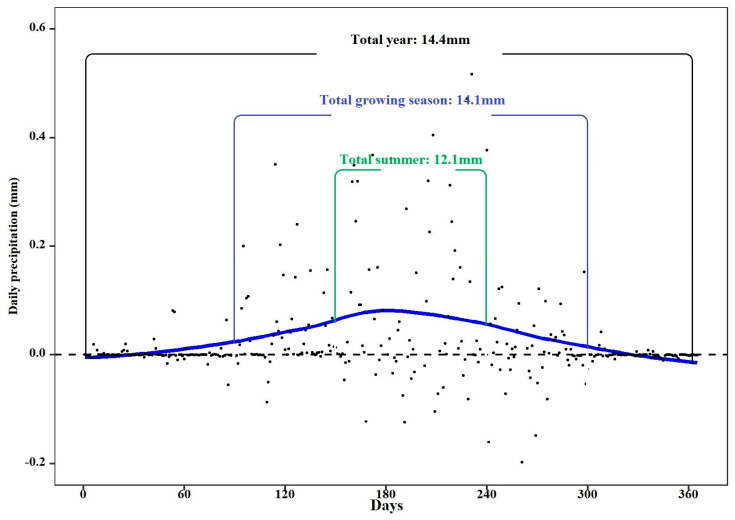
The seasonal variation of precipitation changes following vegetation restoration. Total year (From January to December); Total growing season (From March to October); Total summer (From June to August).

The results based on the WRF model simulations indicate that vegetation restoration promotes P, with the magnitude of annual P increased by 14.4 mm. The P increase mainly occurred during the growing season (14.1 mm), especially in summer (12.1 mm), while the impact on P in the non-growing season was less conspicuous (0.3 mm), with P increase in January (0.07 mm), February (0.17 mm), March (0.04 mm), November (0.08 mm) and P decrease in December (−0.03 mm) (Figure 3).

**Figure 4:**
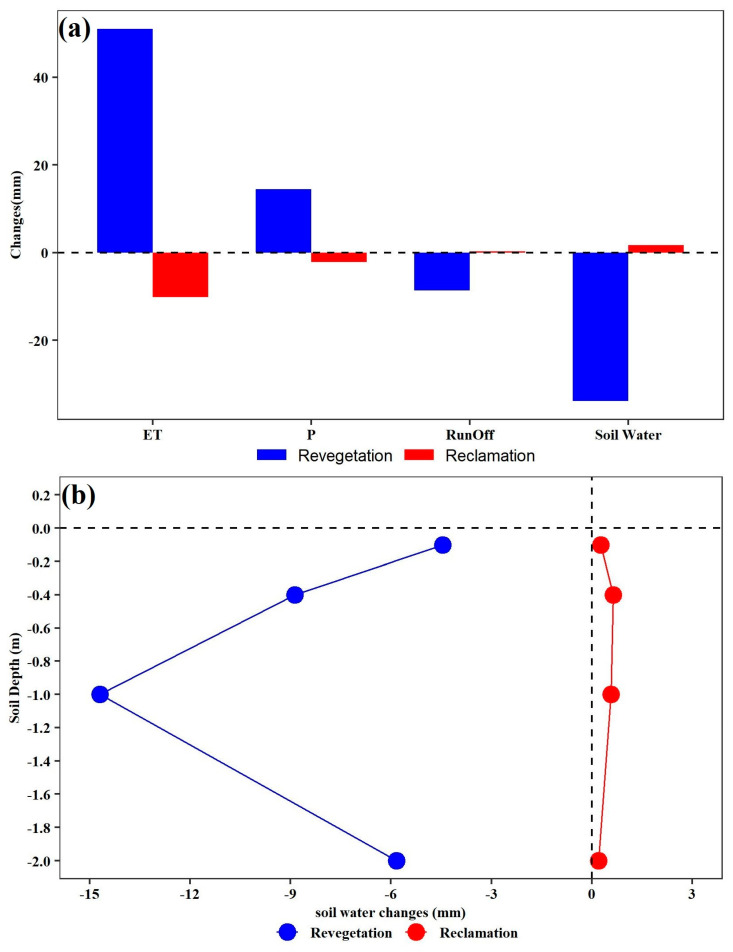
The effect of revegetation and reclamation on evapotranspiration (ET), precipitation (P), runoff, and soil water (**a**); The effects of revegetation and land reclamation on soil water content at various soil depths (0–10 cm, 10–40 cm, 100 cm, and 200 cm) within the Loess Plateau region (**b**).

Revegetation markedly increased ET by 51.0 mm a^−1^ and reduced runoff by 8.6 mm a^−1^. At a depth of two meters below the surface in the simulation, there was a noticeable change in soil water content (Figure 4a). Revegetation decreased soil water content by 33.9 mm a^−1^, with the magnitude of the soil water deficit increasing with soil depth to a maximum value of −14.7 mm a^−1^ at a soil depth of 40–100 cm and then declining (Figure 4b).

**Figure 5:**
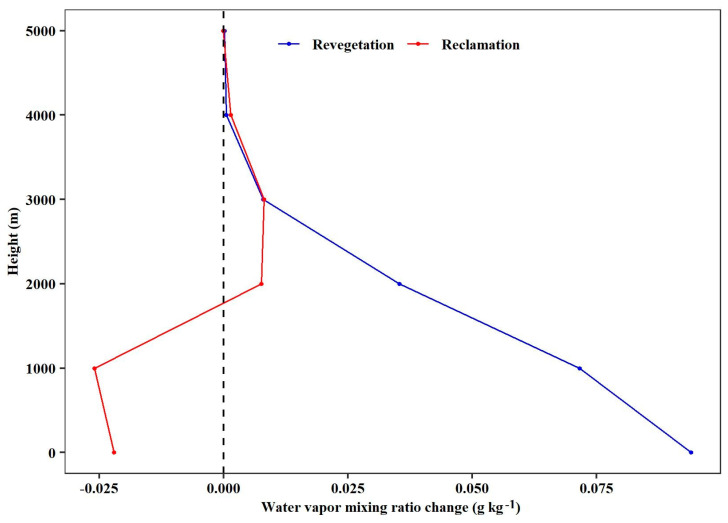
The effect of revegetation and reclamation on atmospheric water content for different heights above the ground over the Loess Plateau.

Vegetation changes not only impact surface hydrological processes but also influence the variability of atmospheric water content, with the primary effects occurring within the atmospheric boundary layer (from the Earth’s surface to a height of 2000 m). These findings indicate that vegetation restoration led to an increase in atmospheric water content of 1.3%. Furthermore, the effect varies with height, being larger and closer to the ground. Near the surface, there is a notable increase of 0.09 g kg^−1^ (2.0%) in atmospheric water content (Figure 5).

### 3.2. The Hydrological Effects of Reclamation

As the issue of soil drying in the LP intensifies, scholars are increasingly questioning the effectiveness of GFGP. If a policy of land reclamation was to be implemented instead of GFGP, what effect would it have on the hydrological processes in the LP? In contrast to revegetation, land reclamation had the effect of decreasing annual P: it fell by 2.2 mm (Figure 4a).

Land reclamation markedly reduced ET by 10.2 mm and increased runoff by 0.3 mm (Figure 4a). The simulations reveal a notable increase in soil water content (1.7 mm) at a depth of two meters below the surface due to land reclamation. The magnitude of this soil water content increase varied with soil depth, initially rising and then declining with depth, with the most notable increase in soil water content (0.64 mm) occurring at a depth of 10–40 cm (Figure 4b).

Compared with revegetation, land reclamation led to a decrease in atmospheric water content of 0.2%. This impact decreases with height, but close to the surface, the atmospheric water content decreases by 0.02 g kg^−1^ (−0.5%) (Figure 5).

### 3.3. The Impacts of Vegetation Change on the Hydrological Processes

**Figure 6:**
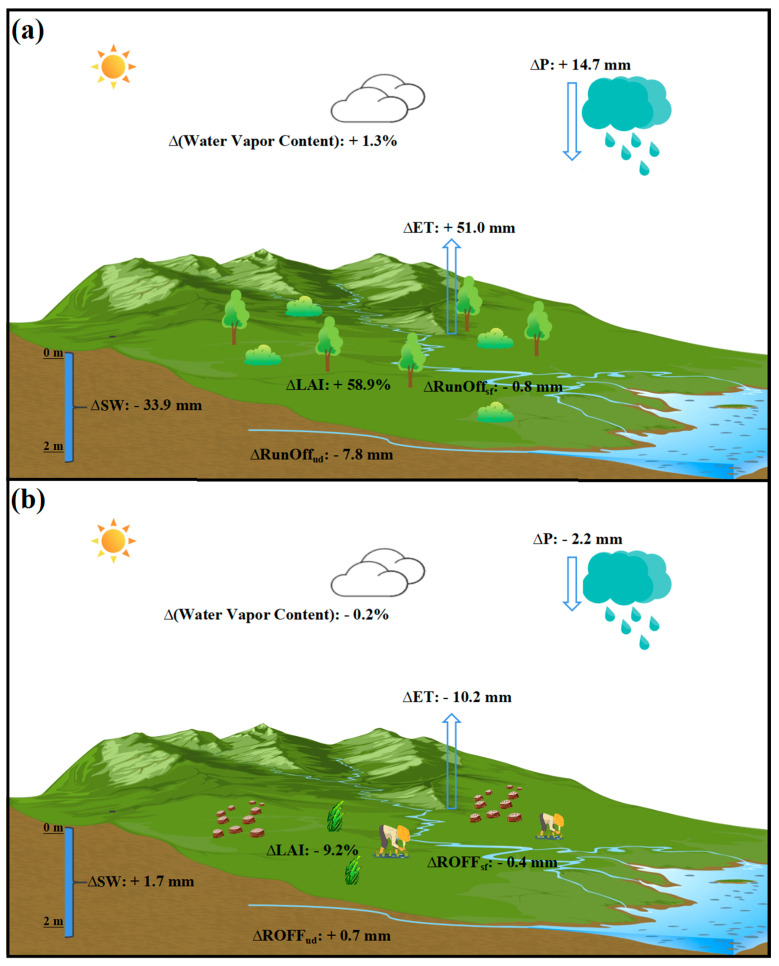
A schematic diagram showing changes in hydrological processes following revegetation (**a**) and reclamation (**b**) derived by regional climate modeling with WRF. Changes are shown for annual precipitation (P), leaf area index (LAI), atmospheric water vapor content, evapotranspiration (ET), surface runoff (ROFF_sf_), underground runoff (ROFF_ud_), and soil water content (SW).

The changes in land surface hydrological processes and the associated feedbacks in atmospheric water content and P, which underlie the observed hydrological effects following vegetation changes, are depicted in a schematic diagram (Figure 6) and table (Table S3). Overall, vegetation LAI increased by 59% from 2001 to 2019, resulting in an increase in annual ET of 48.9 mm, which led to an increase in atmospheric water content (1.3%) and P (14.7 mm). The enhanced ecosystem ET consumes a large number of surface water resources, leading to a reduction in runoff (surface runoff: −0.8 mm, underground runoff: −7.8 mm) and soil water content (−33.9 mm) (Figure 6a).

In contrast to revegetation, land reclamation led to a slight reduction in LAI by 9.2%, which in turn resulted in a 10.2 mm decrease in annual ET. This decrease caused a reduction in atmospheric water content of 0.2% and a decrease in P of 2.2 mm. The decrease in P results in reduced surface runoff (−0.4 mm). The weakened ecosystem ET reduces the consumption of groundwater resources, thereby increasing underground runoff by 0.7 mm and soil moisture by 1.7 mm (Figure 6b).

## 4. Discussion

Vegetation restoration enhances P and ET, leading to an increase in atmospheric water content, particularly during the growing season, a finding that is consistent with most previous studies. Numerical experiments performed over the whole of LP in previous studies have demonstrated that revegetation can significantly augment rainfall and ET [18,36,37,47,60]. The underlying mechanism is that vegetation greening can generally facilitate surface ET due to the larger leaf area, an aerodynamically rougher surface, and higher canopy conductance for transpiration [20,23,24,25,26], resulting in a more humid atmosphere and, consequently, increased P.

However, increased P due to revegetation over the LP was not enough to compensate for the increase in ET, resulting in diminished runoff and soil water content. Previous studies have also demonstrated that an increase in ET following the initiation of revegetation markedly surpasses accompanying increases in P, leading to a considerable reduction in runoff [9,20] and serious soil desiccation [27,61,62], threatening the region’s water resources [6,20,40,63]. The underlying mechanism here is that revegetation facilitates enhanced ET, but the corresponding increase in P is not large enough to compensate for it, intensifying the excessive demand for groundwater (runoff and soil water content). This unsustainable water demand lowers water availability for agriculture or other human demands [13,14,15,40,64].

However, numerical experiments over the whole of LP performed by Zhang et al. [41] led to the conclusion that the increased rainfall due to revegetation over LP was large enough to compensate for the increase in ET and that there was little resultant effect on soil water content. This simulated negligible soil moisture change associated with revegetation is contradicted by extensive studies based on observations [14,27,65]. Several improvements in our WRF simulation over those used in previous studies may explain the discrepancy between their results and those of this study. First, the land surface forcings used in the WRF experiments in previous studies were obtained from multiple data sources (e.g., the land cover dataset is from ESA; the LAI, VEGFRA, and surface albedo datasets are from GLASS), possibly increasing the uncertainty of the model simulations. In order to reduce such modeling uncertainty caused by using multiple datasets from a variety of sources, in all of our WRF simulations, we incorporated MODIS satellite observations into the WRF model to replace the default land surface forcings for land cover distribution, ground vegetation fraction, LAI and surface albedo. Second, our experiment design effectively eliminates the nonlocal effects of vegetation changes in the surrounding area of the LP. To objectively assess the ecohydrological impacts of revegetation, it is essential to isolate the effects of atmospheric circulation alterations attributed to surface changes beyond the study area. Otherwise, within the context of global climate change, the subtle climate impacts induced by revegetation in the LP may be obscured.

We also investigated the potential impact on the hydrology of the LP if land reclamation was continued in the 2000s, which has not been assessed before but is important for both scientific communities and policymakers. Compared with revegetation, land reclamation can result in a reduction of ET, leading to a subsequent decline in atmospheric moisture content and a decrease in P levels. Decreasing P levels leads to a commensurate decline in surface runoff. The weakened ecosystem’s ET decreases the consumption of groundwater resources, resulting in increased underground runoff and soil water content.

This result suggests that planting trees may have larger negative impacts on surface water availability than planting grains. This is likely due to their larger leaf area, higher aerodynamic roughness, and more developed rooting system, which generally result in trees having a higher water consumption than grains [17,66,67]. Revegetation and land reclamation exhibit different impacts on soil water content levels at different soil depths. Specifically, revegetation predominantly utilizes water from deeper soil strata, while land reclamation primarily depletes the surface soil water. Compared with crops, planted forests have deeper roots, which shift their water source from shallow to deep soils and tend to extract more water from deeper soil [32,68,69,70]. Previous studies have indicated that revegetation has resulted in severe soil water deficits (1037−1531 mm) in deep soil layers in LP [71]. Although land reclamation enhances the availability of groundwater for agriculture or other human demands, it concurrently poses a risk of exacerbating soil erosion in the LP as a result of deforestation. Compared with land reclamation, vegetation restoration can intercept rainfall, reduce its impact energy, and prevent splash erosion. It also slows down and reduces surface runoff, mitigating flood and sheet erosion [72]. Moreover, vegetation restoration can anchor and reinforce soil particles with its spreading root systems and stabilize hillslopes through axial root reinforcement to weaken gravitational erosion [73,74].

The present vegetation over the LP, which to some extent reflects decades of revegetation, may already exceed the limit that the local water supply can support [20,29]. Although revegetation can help to improve the quality of the environment, halt soil erosion, and mitigate warming, if and how to implement further revegetation should be cautiously determined, with the pros and cons of revegetation being carefully weighed for the LP. In consequence, a scientifically determined vegetation composition should be explored to ensure sustainable management of water resources and agriculture.

## 5. Conclusions and Future Work

This study, using a coupled land-atmosphere model, evaluated how the hydrology of the LP has been impacted by revegetation and by reclamation. The findings show that vegetation restoration stimulates an increase in both P and ET, thereby enhancing the atmospheric water content. However, the increase in P does not suffice to counterbalance the heightened ET, thereby causing a decrease in both runoff and soil water content. The vegetation restoration in the LP comes at the cost of over-consumption of soil water. Compared with revegetation, land reclamation has the effect of decreasing annual P: it reduces ET and surface runoff and increases underground runoff and soil water. Although land reclamation increases the underground water availability for agriculture or other human demands, it may aggravate soil erosion on the LP due to deforestation. Our study provides an important scientific basis for guiding current and future revegetation (reclamation) activities in other semi-arid and arid regions around the world towards sustainable ecosystem development and water resources management.

## Data Availability

The data used in the present work have been listed in the Data Sources.

## Supplementary Materials

The following supporting information can be downloaded at: https://www.mdpi.com/article/10.3390/plants13192735/s1, Table S1. Main physical parameterizations used in the WRF simulation. Table S2. The information of experimental design of the WRF simulation. Figure S1. The nonlocal effects of vegetation changes in the area surrounding the Loess Plateau on precipitation within the plateau. Figure S2. The process of constructing a land surface forcings dataset for the EXreclamation simulation. Figure S3. Seasonal variations evaluation of the WRF model precipitation simulation. Table S3. The changes in hydrological processes following revegetation and reclamation derived by regional climate modeling with WRF.
