# Supplementary material for: Vegetation Restoration Increases the Drought Risk on the Loess Plateau
Source: Plants (Basel). 2024 Sep 30;13(19):2735. doi: 10.3390/plants13192735 (PMC11478491; doi:10.3390/plants13192735)
Supplement: Supplementary file 1 [file plants-13-02735-s001.zip › Supplementary materials.pdf]

Supplementary material

Table S1. Main physical parameterizations used in the WRF simulation.

Physical scheme	Information
Land surface model	Noah LSM
Cumulus scheme	Multi-scale Kain-Fritsch (D01:turned off; D02:turned on)
Radiation scheme	RRTMG (longwave) and Dudhia (shortwave)
Microphysics scheme	CAM5.1
PBL scheme	MYNN2.5
Surface layer	MM5 Monin–Obukhov

Table S2. The information of experimental design of the WRF simulation.

Experiment	**Land forcings** (LAI, VEGFRA, Albedo, Land cover)		
	D01	D02	LP
EX_{2001}	2001	2001	2001
EX_{2019}	2019	2019	2019
EX_{2019_S}	2001	2001	2019
$EX_{reclamation}$	2001	2001	Reclamation

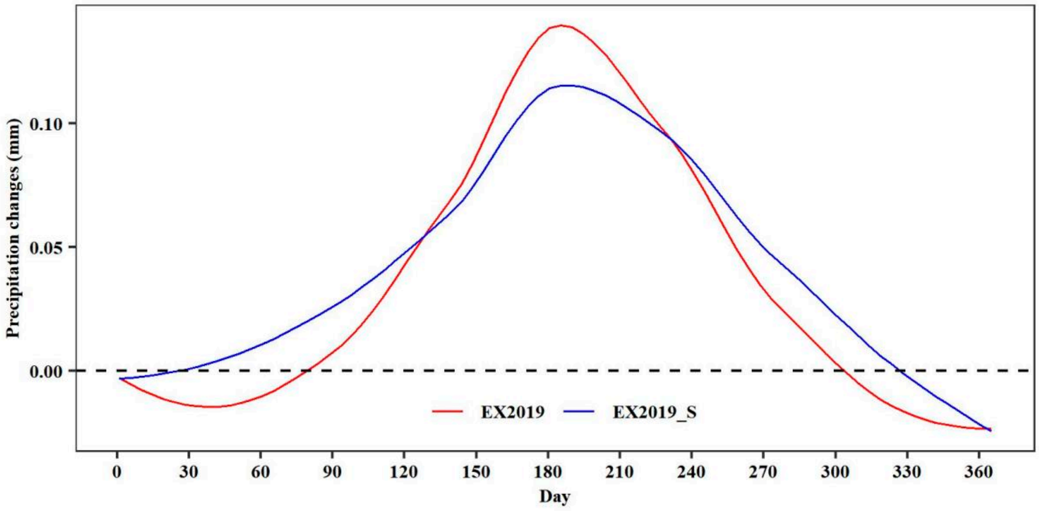

Figure S1. The nonlocal effects of vegetation changes in the area surrounding the Loess Plateau on precipitation within the plateau.

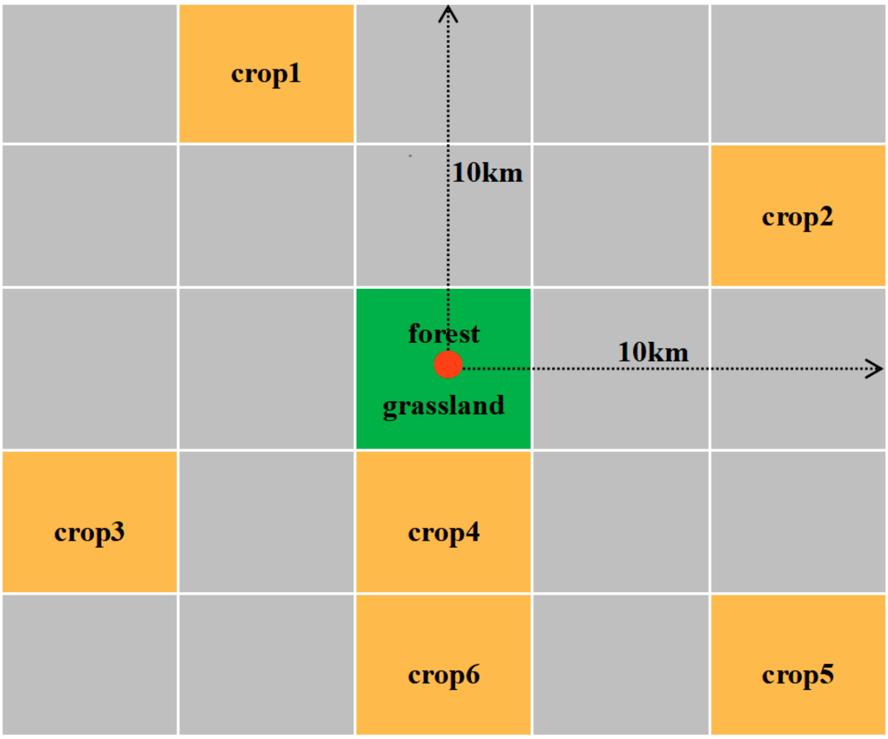

Figure S2. The process of constructing a land surface forcings dataset for the EXreclamation simulation.

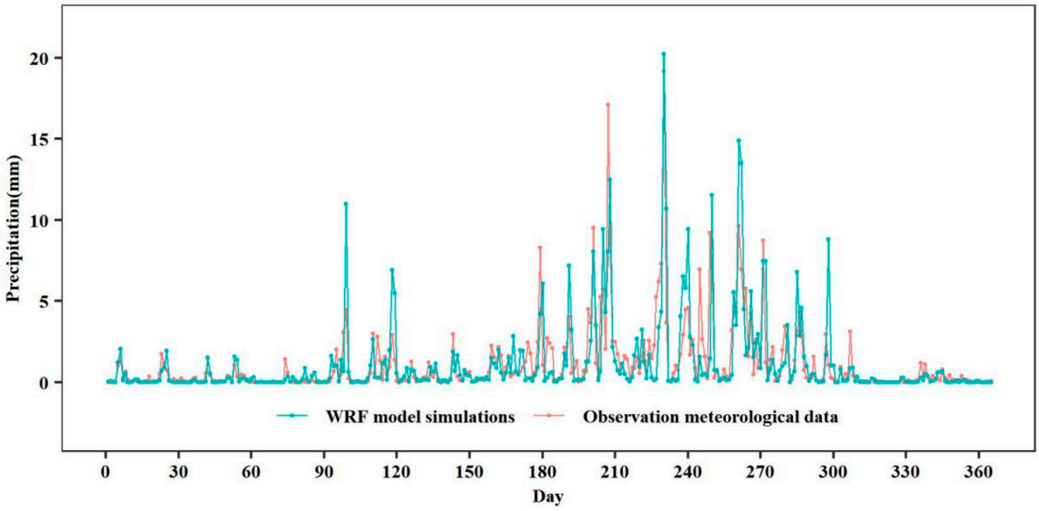

Figure S3. Seasonal variations evaluation of the WRF model precipitation simulation.

Table S3. The changes in hydrological processes following revegetation and reclamation derived by regional climate modeling with WRF.

Experiment	ΔLAI	ΔP	ΔET	ΔSW	$\Delta ROFF_{sf}$	$\Delta ROFF_{ud}$	Δ Water vapor content
Revegetation	+58.9%	+14.7mm	+51.0mm	-33.9mm	-0.8mm	-7.8mm	+1.3%
Reclamation	-9.2%	-2.2mm	-10.2mm	+1.7mm	-0.4mm	+0.7mm	-0.2%
